# Effects of the diphenylheptane extract of *Alpinia officinarum *rhizomes on ethanol-induced gastric ulcers in mice

**DOI:** 10.22038/ijbms.2021.53644.12068

**Published:** 2021-05

**Authors:** Kaiwen Lin, Huijuan Qu, Yinfeng Tan, Tang Deng, Bingmiao Gao, Na Wei

**Affiliations:** 1 School of Pharmacy, Hainan Medical University, Haikou, China; 2 Hainan Provincial Key Laboratory for Research and Development of Tropical Herbs, Haikou, China; 3 Key Laboratory of Tropical Translational Medicine of Ministry of Education, Haikou, China; 4 Intervention Vascular Surgery of First Affiliated Hospital of Hainan Medical University, Haikou, China; 5 Key Laboratory of Emergency and Trauma of Hainan Medical University, Ministry of Education, Haikou, China

**Keywords:** Anti-inflammation, Anti-oxidant, Diphenylheptane, Ethanol, Gastric protection, Gastric ulcer

## Abstract

**Objective(s):**

Experimental studies have revealed that *Alpinia officinarum* Hance (Zingiberaceae) exhibits a gastrointestinal protective effect. The present study aimed to investigate the effects of diphenylheptanes (DPHs) extracted from* A. officinarum* rhizomes on ethanol-induced gastric ulcers in BALB/c mice.

**Materials and Methods:**

A total of 60 female BALB/c mice were divided into six groups as follows: negative control, which received sodium carboxymethyl cellulose; positive control, which received ethanol; treatment control, which received ethanol+ranitidine; ethanol+high dose of DPHs; ethanol+medium dose of DPHs; ethanol+low dose of DPHs. Different doses of DPHs were administered orally once daily for seven consecutive days before the animals were subjected to ethanol-induced gastric ulcers.

**Results:**

Various doses of DPHs significantly reduced Gastric ulcers index when compared with the positive control. DPHs treatments and treatment control increased the activity of superoxide dismutase; decreased the levels of inflammatory mediators, malondialdehyde, motilin, and gastrin; decreased the activity of inducible nitric oxide synthase and cyclooxygenase-2; and inhibited the expression of Toll-like receptor 4, myeloid differentiation factor 88, and nuclear factor-κB at the protein and mRNA levels. In addition, DPHs inhibited the expression of transient receptor potential vanilloid 1, calcitonin gene-related peptide, and increased the expression of substance P at the protein and mRNA levels.

**Conclusion:**

The protective effect of DPHs extracted from* A. officinarum* rhizomes against ethanol-induced gastric damages in mice suggests that the extract can be used as an auxiliary supplement for the prevention and treatment of gastric ulcers.

## Introduction

Gastric ulcers is one of the gastrointestinal diseases with a high incidence among clinical diseases. The pathogenesis of gastric ulcers is complex and multifactorial, and it is principally caused by the imbalance between the defense factors of the body such as bicarbonate (1), prostaglandin (2), nitric oxide (3), and aggressive factors in the gastric mucosa such as gastric acid (4) and pepsin (5). A few studies have revealed that exogenous causes, such as long-term use of non-steroidal anti-inflammatory drugs (NSAIDs) (6, 7), excessive alcohol abuse (8), mental stress (9), and *Helicobacter pylori* infection (10), can also lead to gastric ulcers. Ethanol-induced gastric ulcers is predominantly associated with inflammation and oxidative stress, which are characterized by hemorrhagic plaque and extensive erosion of the gastric mucosa, in addition to the production and release of numerous pro-inflammatory cytokines and oxygen free radicals (11), and accumulation of immune cells in the inflamed area. These, in turn, aggravate gastric mucosal injury and tissue edema, erosion, and hyperemia, causing abdominal pain, gastric perforation, and gastric bleeding (12,13). In the treatment of gastric ulcers, several drugs including proton pump inhibitors and histamine type-2 (H2) receptor antagonists have drawbacks such as poor overall efficacy, large adverse reactions, and easy recurrence after drug withdrawal (14). Therefore, it is crucial to identify an efficient drug for the treatment of gastric ulcers, and studies have revealed that medicinal plants are effective in the treatment of clinical gastric ulcers, resulting in fewer adverse reactions and a low recurrence rate (15, 16).

*Alpinia officinarum* is a perennial herb, which is often used as a dietary supplement, in food spices, and as a decoction to prevent and improve the treatment of functional gastrointestinal diseases. The herb is primarily composed of volatile oils, flavonoids, and diphenylheptanes (DPHs) (17). In particular, DPHs are considered the most bioactive components in the rhizomes of *A. officinarum *(17). The present study aimed to elucidate the preventive, therapeutic, and protective effects of DPHs extracted from *A. officinarum* rhizomes on gastric ulcers, and to explore the anti-inflammatory and analgesic effects on gastric ulcers. We established an ethanol-induced gastric ulcers mice model to evaluate the efficacy and potential mechanisms of DPHs in gastric ulcers to provide a theoretical basis for verifying the protective effects of DPHs on gastric ulcers and the potential use of the extract as a functional nutritional auxiliary drug for the prevention and treatment of gastric ulcers.

## Materials and Methods

***Plant materials and extraction***

Samples of *A. officinarum* rhizomes were obtained from Haikou City, Hainan Province, China, in October 2017. The botanical identity of the plant samples was confirmed by professor Niankai Zeng of Hainan Medical University (voucher No. 20171024). The voucher specimens were deposited in the herbarium of the Natural Pharmaceutical Chemistry of Hainan Medical University. 

***Preparation of plant extracts and liquid chromatography tandem-mass spectrometry analysis***

The DPH extracts from *A. officinarum* rhizomes were prepared according to a method previously developed by our research group (18). Fresh rhizomes of *A. officinarum* (1 kg) were air-dried at 60 °C for three days, weighed, and subsequently pulverized. The powder was extracted using a Soxhlet apparatus and refluxed with eight-fold 80% ethanol for 1 hr. Samples were extracted twice under the same conditions. The ethanol extracts were mixed and concentrated to 40% under reduced pressure in a rotary evaporator. Afterward, the extract was purified on an AB-8 macroporous adsorption resin column with 80% ethanol. The eluted fraction was subjected to silica gel column chromatography and eluted on a petroleum ether-ethyl acetate gradient to obtain five fractions, with components one, two, and five subjected to silica gel column chromatography, and the product was subsequently eluted with methanol to obtain fraction A (DPHs). We analyzed the chemical constituents of DPHs extracted from *A. officinarum* by LC–MS/MS according to a previously reported method (19).

***Experimental animals***

A total of 60 six-week-old female BALB/c mice weighing 18–22 g were procured from Tianqin Biotechnology, Changsha, China. The mice were housed under controlled conditions (12 hr light/dark cycle, room temperature of 24 ± 1 °C, and relative humidity of 40–60%), and were allowed to freely access a standard certified rodent diet and tap water. All mice experiments were handled per the Guide for Care and Use of Laboratory Animals, and the requirements of the Animal Experimental Ethics Committee, Hainan Medical University, Haikou, China. 

***Animal experimental design***

After three days of adaptive feeding, the mice were randomly divided into six groups with ten mice in each group as follows: G1: negative control, which received sodium carboxymethyl cellulose (Na-CMC); G2: positive control, which received ethanol; G3: treatment control, which received ethanol+ranitidine (RAN) (100 mg/kg); G4: ethanol+high dose of DPHs (126.8 mg/kg); G5: ethanol+medium dose of DPHs (63.4 mg/kg); G6: ethanol+low dose of DPHs (31.7 mg/kg). RAN and DPHs were formulated with 0.5% Na-CMC and administered intragastrically for seven days. Mice were subjected to fasting for 12 hr before administration of the last treatment, although free access to drinking water was allowed. Acute gastric injury was induced through intragastric administration of a single dose of 100% ethanol (10 ml/kg) 2 hr after a final treatment administration. All mice were euthanized under deep isoflurane anesthesia 1 hr after ethanol induction. The gastric tissues of mice were rapidly removed, cut along the great curvature of the stomach, and rinsed with cold normal saline. The gastric injury site was photographed with a digital camera, and the gastric ulcers index and inhibition rates of gastric ulcers drug were evaluated according to the method of determining hemorrhagic mucosal lesion area of gastric tissues (formulas [1] and [2]). Finally, the stomach was divided into two parts: one part was placed in 4% paraformaldehyde solution for subsequent histopathological analyses, and the other part was stored in a –80 °C refrigerator. Afterward, biochemical analyses were carried out.

***The gastric injury index and preventive inhibition***

The ulcer index (UI) was calculated according to Equation (1) as follows: 

Ulcer Index (UI) = (1×A) + (2×B) + (3×C)          [1]

where A is the number of small ulcers, B is the number of medium ulcers, and C is the number of linear ulcers; the size of ulcers is as follows: small ulcer ≤ 1 mm; 3 mm ≥ medium ulcer >1 mm; linear ulcer > 3 mm. 

The percentages of DPHs and RAN group inhibition were calculated according to the following equation (2).

Inhibition = ((UI^a^ - UI^b^) × 100%)/UI^a^           [2]

where UI^a^ is the ulcers index of the ethanol group, and UI^b^ is the ulcers index of the DPHs or RAN group.

***Histopathological analysis***

Gastric tissues excised from the six experimental groups were fixed in 4% paraformaldehyde solution for 48 hr. Tissues were dehydrated with an increasing series of ethanol solutions and xylene and embedded in paraffin. Paraffin sections were subsequently sliced into thin slices with a thickness of 3 μm. The tissues were deparaffinized and subsequently stained with hematoxylin-eosin for histological evaluation.

***Enzyme-linked immunosorbent assay***

The levels of tumor necrosis factor-alpha (TNF-α), interleukin 1 beta (IL-1β), interleukin 6 (IL-6), prostaglandin E2 (PGE2), nitric oxide (NO), and malondialdehyde (MDA) were determined using an ELISA kit (Elisa Biotech, Shanghai, China). The activities of SOD, iNOS, and COX-2 were measured simultaneously according to the manufacturer’s instructions (Nanjing Jiancheng Bioengineering Institute, Nanjing, China). The absorbance values were measured on an Epoch Micro Plate Spectrophotometer at a wavelength of 450 nm (BioTek Epoch; BioTek Instruments Inc., Winooski, VT, USA).

***Western blot analysis***

Proteins were collected and their concentrations determined using a bicinchoninic acid (BCA) protein assay kit (Beyotime, Nanjing, China). Equal amounts of proteins (50 μg) from each sample were resolved by sodium dodecyl sulfate-polyacrylamide gel electrophoresis (SDS–PAGE), and the electrophoretically resolved proteins were transferred onto polyvinylidene fluoride (PVDF) membranes (Millipore Sigma, Burlington, MA, USA). The membranes were blocked with 5% non-fat dry milk for 1 hr on a shaker and then incubated overnight at 4 °C with primary anti-toll-like receptor 4 (TLR4; 1:1000 dilution; Abcam, cat. no. ab22048), myeloid differentiation factor 88 (MYD88; Abcam, cat. no. Ab219413), interleukin-1 receptor-associated kinase 1 (IRAK-1; Abcam, cat. no. Ab238), phosphorylated-IκBα (p-IκBα; Abcam, cat. no. Ab133462), NF-κBp65 (Abcam, cat. no. Ab16502), transient receptor potential vanilloid 1 (TRPV1; Abcam, cat. no. ab203103), calcitonin gene-related peptide (CGRP; Abcam, cat. no. Ab81887), substance P (SP; Abcam, cat. no. Ab14184), and β-actin (Abcam, cat. no. Ab8245). The membranes were washed three times with TBST and subsequently incubated with horseradish peroxidase-conjugated secondary antibody (Abcam, cat. no. Ab6721) on a shaker at room temperature (24 ± 1 °C) for 2 hr. Finally, specific bands were detected by a gel imaging system (Bio-Rad, Hercules, CA, USA) using ECL chemiluminescence detection reagents (Beyotime, Nanjing, China). Quantification analysis of protein bands was performed using ImageJ software (National Institutes of Health, USA).

***Determination of mRNA expression levels***

Total RNA was extracted from gastric tissues using Eastep® Super Total RNA Extraction Kit (YEASEN, Shanghai, China). Total RNA was reverse transcribed using Hifair^®^II 1^st^ Strand cDNA Synthesis SuperMix (YEASEN, Shanghai, China) after determination of total RNA concentration using an enzyme labeling instrument. The prepared cDNA was stored at –20 °C as a quantitative polymerase chain reaction (q-PCR) template. PCR was performed using a Hieff^®^ q-PCR SYBR^®^ Green Master Mix Kit (YEASEN, Shanghai, China). The total volume of the reaction system was 20 μl (cDNA template [2 μl]), upstream and downstream primers (0.4 μl), Green Master Mix (6 μl), and acid-free water (11.2 μl). A q-PCR amplification procedure was conducted using a three-step method: pre-denaturation at 95 °C for 5 min (1 cycle); denaturation at 95 °C for 10 sec; and annealing/extension at 60 °C, and extension for 30 sec (a total of 40 cycles). Afterward, the samples were subjected to quantitative real-time PCR analysis using an Mx 3005p qPCR System (Agilent Technologies, Santa Clara, CA, USA), and the results were analyzed based on the cyclic threshold (CT) method. All primers were designed manually (Table 1). 

***Statistical analysis***

Experimental data were expressed as means±standard deviation, and statistical analyses were performed using IBM SPSS Statistics version 25.0 (IBM Corp., Armonk, NY, USA). One-way analysis of variance (ANOVA) and Student’s *t*-test were used to compare means between the experimental groups. A *P*-value<0.05 indicated statistical significance.

## Results

***Identification and analyses of chemical constituents of fraction A (DPHs)***

**Figure 1:**
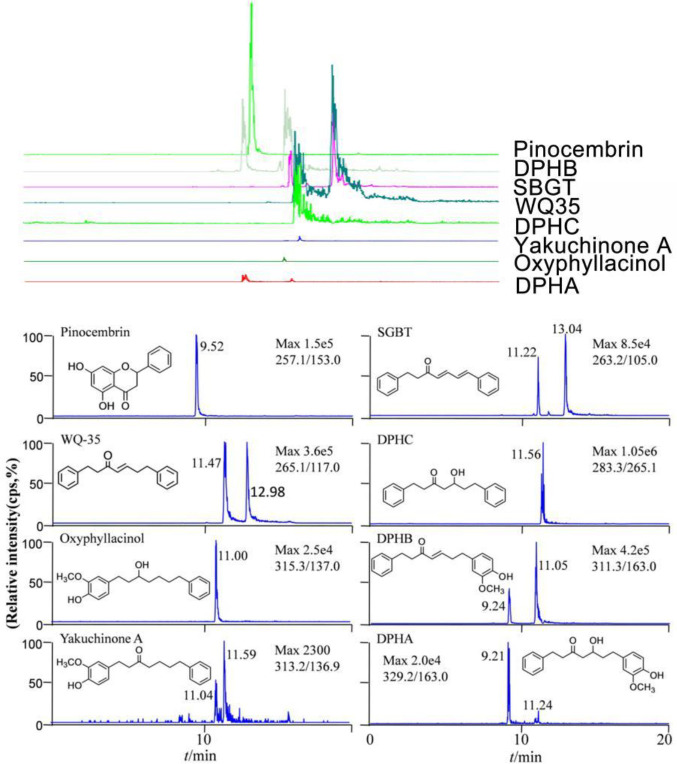
The structure of the eight main components of DPHs

Eight key components were identified, which included Pinocembrin; 1,7-diphenyl-4,6-dien-3-heptanone (SBGT);

1-diphenyl-4-en-3-heptanone (WQ35); 5-hydroxy-7-(4-hydroxy-3-methoxyphenyl) (DPHA); 7-(4-hydroxy-3-methoxyphenyl)-1-phenyl (DPHB); 1,7-diphenyl-5-hydroxy-3-heptanone (DPHC); and oxyphyllacinol. In addition, the signal of yakuchinone-A was detected, but the retention time was not consistent with that of the control substance; therefore, we presumed that it could be a Yakuchinone A isomer. Seven compounds were DPHs, except chosin. The LC-MS/MS spectra of the chemical compounds are presented in Figure 1.

***Effect of DPHs on gastric ulcers index***

**Figure 2:**
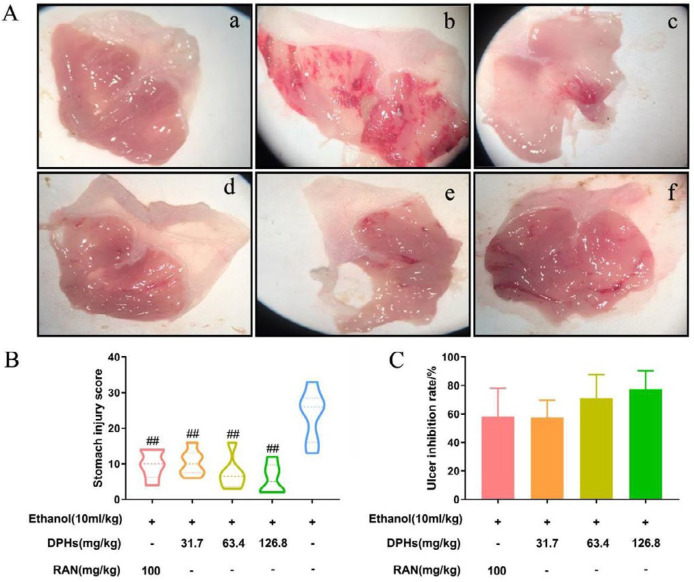
General evaluation of DPHs effects on ethanol-induced gastric mucosal damage. (a) G1, gastric mucosal integrity; (b) G2; (c) G3; (d) G4; (e) G5; (f) G6 showed relief of gastric injury. Compared with G1, G2 showed acute gastric mucosal injury, rough surface, a large number of ulcerative plaques, and erosion, while the gastric tissue pretreated with drugs (G3, G4, G5, and G6) showed significant improvement in gastric injury. (B) ulcer injury index of each experimental group. (C) treatment group inhibited the injury rate. As shown in Figures B and C, with the increase of DPHs concentration, the ulcer index decreased and the ulcer inhibition rate increased. ^*^P<0.05, compared with G1; ^#^*P*<0.05 and ^##^*P*<0.01, compared with G2. The numerical value is expressed as mean ±standard deviation (n=6)

The gastric mucosa of mice in G1 was intact and there was no indication of mucosal erosion. G2 mice exhibited extensive hemorrhagic necrosis and ulcerative plaque formation. DPHs significantly reduced the degree of gastric mucosal injury induced by ethanol in G4, G5, and G6 mice or the RAN group (G3) when compared with G2 mice (Figure 2). A significant inhibition in gastric ulcers index was observed in mice from all DPHs-treated groups (G4, G5, and G6; *P*<0.01) and in mice of the RAN group (*P*<0.05) when compared with mice in G2. The inhibition rates of the gastric ulcers index in mice groups that received high, medium, and low doses of DPHs (G4, G5, and G6) and in G3 mice were 71.04%, 71.33%, 57.64%, and 58.09%, respectively. 

***Histopathological analysis***

**Figure 3:**
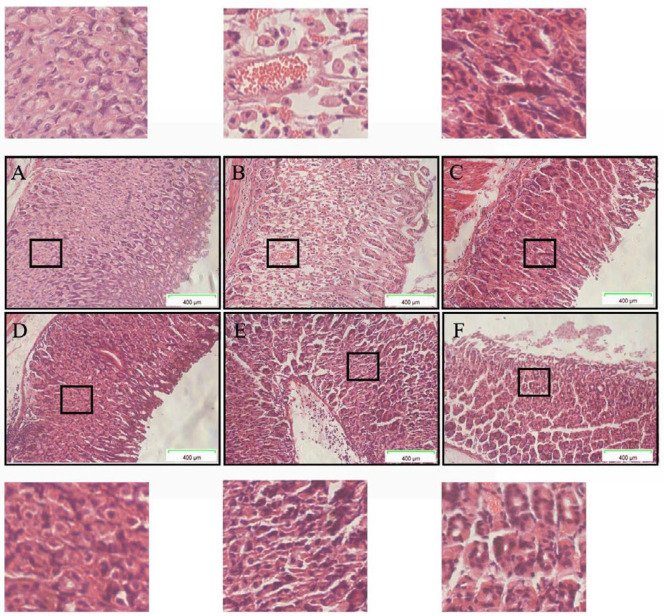
Histopathological features of gastric tissue of mice (hematoxylin-eosin staining (magnification: 400×)) (n=6). (A) G1; (B) G2; (C) G3; (D) G4; (E) G5; (F) G6. The upper left corner of each picture is a local (black wireframe) enlarged map, which shows that G1 has complete tissue structure, neatly arranged cells, and no gastric mucosal injury, while G2 shows incomplete gastric structure and tissue shedding, a large number of inflammatory cells (red granules) infiltrating tissue, cell shrinkage, and enlarged glandular spacing. Compared with G2, the DPHs group showed improved tissue structural integrity, mild mucosal injury, and less inflammatory cell accumulation and infiltration. G4 in the high dose group was more effective in improving acute injury induced by ethanol than middle dose G5 and low dose G6

Histopathological evaluation of gastric tissues revealed that G1 had an intact tissue structure without erosion, ulcers, or inflammatory cell infiltration (Figure 3). Conversely, ethanol stimulation caused marked mucosal ulcers and erosion, irregular arrangement of epithelial cells, destruction of tissue structure, and accumulation of numerous inflammatory cells. The high (G4), medium (G5), and low (G6) doses of DPHs groups and the RAN group (G3) exhibited a reduction in gastric mucosal erosion and inflammatory cell infiltration when compared with G2. 

***Effect of DPHs on the levels of IL-1 β, IL-6, and TNF-α in gastric tissues of mice***

**Figure 4:**
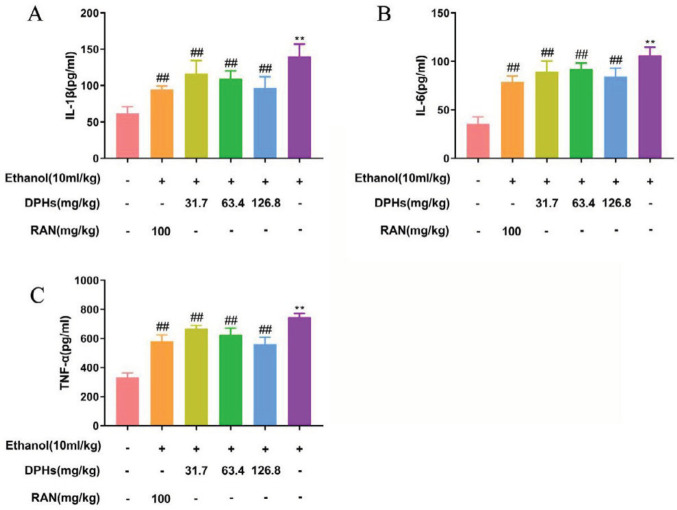
Effect of DPHS on inflammatory mediators in gastric tissue of mice. (A) The level of IL-1β in gastric tissue, (B) The level of IL-6 in gastric tissue, (C) The level of TNF-α in gastric tissue. Values are expressed as means ± standard deviation (n=8). ^**^P<0.01, compared with G1;^ ## ^*P*<0.01, compared with G2

DPHs: diphenylheptanes; PGE2: prostaglandin E2

The levels of TNF-α, IL-1β, and IL-6 in G2 mice significantly increased when compared with corresponding levels in G1 mice (*P*<0.01, Figure 4). The release of inflammatory mediators, TNF-α, IL-1β, and IL-6 in gastric tissues of mice in G4, G5, and G6 (DPHs-treated groups) was significantly inhibited (*P*<0.01). 

***Effects of DPHs on the levels of PGE2 and NO, and the activities of COX-2 and iNOS in gastric tissues of mice***

The levels of PGE2 and NO, and the activities of COX-2 and iNOS in G2 mice were significantly increased when compared with G1 mice (*P*<0.01, Table 2). The increase in the levels of PGE2 and NO, and activities of COX-2 and iNOS in gastric tissues of mice in G4, G5, and G6 (DPHs-treated groups) was inhibited (*P*<0.01).

***Effects of DPHs on the levels of malondialdehyde, motilin, gastrin, and superoxide dismutase activity in gastric tissues***

**Figure 5:**
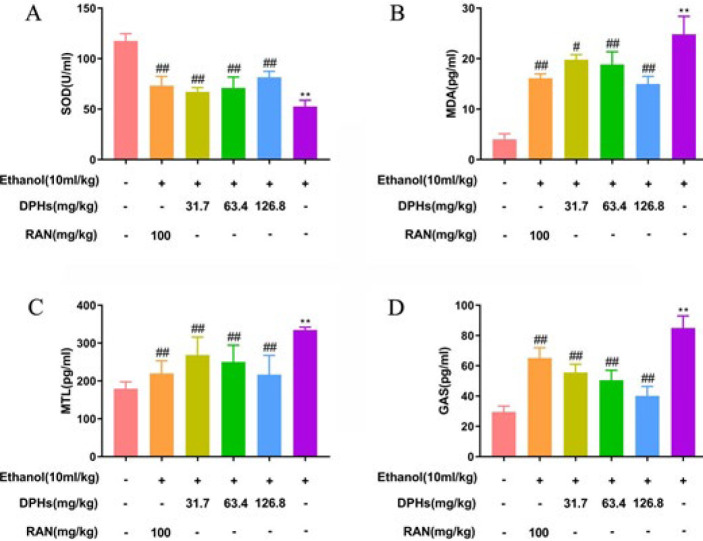
Effects of DPHS on MDA, MTL, GAS and SOD contents in gastric tissues. (A)The activity of SOD in gastric tissue, (B) The level of MDA in gastric tissue, (C) The level of MTL in gastric tissue, (D) The level of GAS in gastric tissue.Values are expressed as means±standard deviation (n=6). ^**^P<0.01, compared with G1; ^#^*P*<0.05 and ^##^*P*<0.01, compared with G2

DPHs: diphenylheptanes; MDA: malondialdehyde; MTL: motilin; GAS: gastrin; SOD: superoxide dismutase

The effect of DPHs on superoxide dismutase (SOD) activity and MDA levels in gastric tissues are presented in Figure 5. Ethanol stimulation decreased SOD enzyme activity (*P*<0.01, Figure 5A) and increased MDA levels in G2 gastric tissues when compared with G1 gastric tissues (*P*<0.05; *P*<0.01, Figure 5B). The effect of the DPHs-treated group was similar to that of the RAN group. The high (G4), medium (G5), and low (G6) doses of DPHs groups significantly reduced MDA levels and increased SOD enzyme activity in gastric tissues; however, a DPH dose of 126.8 mg/kg was slightly more effective than that of RAN. As shown in Figure 5, motilin (MTL) (*P*<0.01, Figure 5C) and gastrin (GAS) (*P*<0.01, Figure 5D) levels in ethanol-treated (G2) mice were significantly higher than the corresponding levels in G1 mice, while MTL and GAS levels in the DPHs-treated (G4, G5, and G6) and RAN (G3) groups decreased.

***Treatment with DPHs significantly suppressed the activation of TLR4/MYD88/NF-κB***

**Figure 6:**
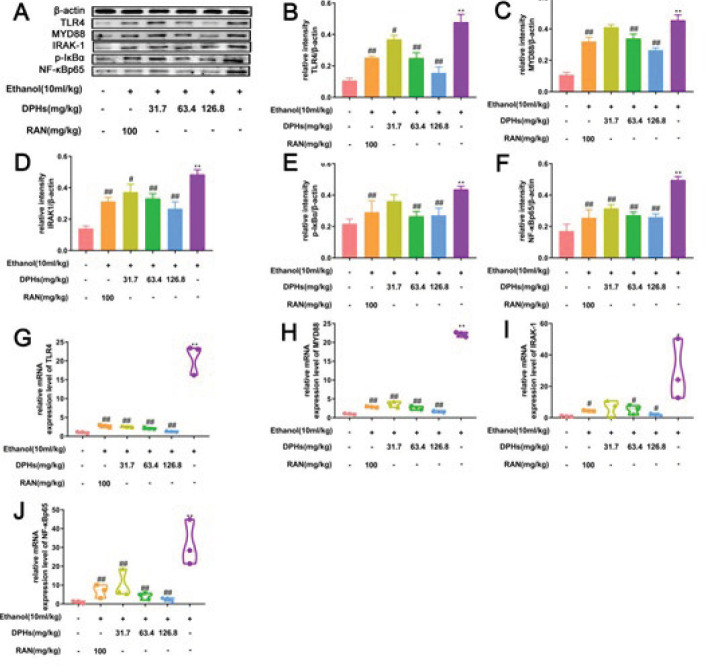
(A) Effects of DPHs on the proteins expression of TLR4, MYD88, IRAK-1, p-IκBα, and NF-κBp65 in gastric tissues. (B-F) Quantification of the relative proteins levels of TLR4, MYD88, IRAK-1, p-IκBα, and NF-κBp65. (G-J) Gene expression level of TLR4, MYD88, IRAK-1, and NF-κBp65. ^**^P<0.01, compared to the G1; ^#^*P*<0.05 and ^##^*P*<0.01, compared to the G2

TLR4: toll-like receptor 4; MYD88: myeloiddifferentiationfactor88; IRAK1: interleukin-1 receptorassociated kinase 1

We detected the expressions of TLR4, MYD88, IRAK-1, p-IκBα, and NF-κBp65 proteins in gastric tissues of mice to determine the effect of DPHs on the TLR4/MYD88/NF-κB signaling pathway. The expression of related proteins in G1 gastric tissues was low, and ethanol significantly increased the expressions of TLR4, MYD88, IRAK-1, p-IκBα, and NF-κBp65 proteins in G2 gastric tissues (Figure 6). However, treatment with DPHs (G4, G5, and G6) and RAN (G2) decreased the expressions of TLR4, MYD88, IRAK-1, p-IκBα, and NF-κBp65 proteins in gastric tissues (*P*<0.05 and *P*<0.01). Similarly, we observed that the gene expression levels of TLR4, MYD88, IRAK-1, and NF-κBp65 were consistent with the protein assay results (*P*<0.05 and *P*<0.01; Figure 6).

***Effect of DPHs on expressions of TRPV1, CGRP, and SP at the protein and mRNA levels in gastric tissues of mice***

Western blot analysis revealed that expression levels of TRPV1 and CGRP proteins were up-regulated, whereas expression levels of SP proteins were down-regulated in ethanol-treated (G2) mice. Treatment of mice with DPHs (G4, G5, and G6) inhibited expressions of TRPV1 and CGRP proteins and promoted up-regulation of SP proteins. The results of real-time fluorescence-based PCR analysis were consistent with those of the protein experiment, which indicated that expression levels of TRPV1, CGRP mRNA, and the proteins induced by ethanol decreased while expression levels of SP mRNA and proteins increased in all DPHs-treated groups (G4, G5, and G6).

## Discussion

Treatment of gastric ulcers using traditional medicine is achieved by two types of antisecretory drugs (H2 receptor antagonists such as RAN and proton pump inhibitors such as omeprazole) or combined with antibiotics on the premise that previous drugs were used to treat gastric ulcers caused by *H. pylori* (20, 21). However, the side effects of the traditional drugs cannot be overlooked, and recent studies have revealed that dietary herbs are comparable to or superior to other drugs, such as omeprazole or cimetidine, and present fewer side effects (22, 23). In the present study, we evaluated the protective effects of *A. officinarum* on ethanol-induced gastric ulcers in mice and attempted to determine the anti-inflammatory and gastroprotective effects of DPHs extracted from rhizomes of *A. officinarum*. We successfully established a mouse model of ethanol-induced gastric ulcers and used it to evaluate the gastroprotective effects of DPHs. Our results reveal that: (1) DPHs can reduce gastric ulcers index, improve gastric mucosal injury, and protect mucosal integrity in mice; (2) DPHs can ameliorate ethanol-induced gastrointestinal dysfunction and inhibit excessive secretion of GAS and MTL in mice; (3) DPHs can inhibit ethanol-induced and oxidative stress-induced gastric ulcers; (4) DPHs can inhibit the secretion and release of downstream pro-inflammatory mediators, TNF-α, IL-1β, IL-6, NO, PGE2, COX-2, and iNOS by inhibiting the expression of TLR4/MYD88/NF-κB proteins; (5) ethanol induces activation of TRPV1 signal in mice gastric tissues and mediates the occurrence of neuropathic pain, while DPHs inhibit activation of TRPV1, and the release and effect of sensory neuropeptides (SP, CGRP). Such effects collectively reduce ethanol-induced gastric mucosal injury and prolongation of pathological reactions.

Ethanol-induced gastric ulcers presents through a series of cascade reactions. First, gastric mucosa is stimulated by ethanol resulting in numerous blood spots and erosion, and persistent injury leads to disorders associated with the secretion of gastrointestinal hormones (including GAS and MTL) in gastric tissues and damage of microvessel endothelial cells, which in turn, affects blood flow and oxygen supply in gastric mucosal tissues, oxidative stress, mucosal injury and necrosis, and further aggravation of gastric ulcers (24-27). In the present study, we observed that ethanol stimulation eroded gastric tissues and caused acute damage to the gastric mucosa in mice, including hemorrhagic erosion of the gastric mucosa and diffuse ulcers (Figures 2A and B), destruction of gastric mucosal structural integrity, mucosal edema, and inflammatory cell infiltration (Figure 3B), which demonstrated the successful establishment of our ethanol-induced acute gastric ulcers mouse model (28-30). Also, the disorders associated with gastrointestinal function caused by increases in GAS and MTL secretion in the ethanol-treated group (G2) can be used as an indicator of successful establishment of gastric ulcers when compared with the negative control group (G1) (31). Furthermore, the study revealed that MDA levels in G2 gastric tissues significantly increased and SOD enzyme activity decreased, which was consistent with the findings of Almasaudi and Rami *et al*. (32, 33). Ethanol can increase MDA levels in gastric tissues and decrease SOD enzyme activity, which suggests that ethanol stimulation can reduce the ability of gastric mucosa to resist oxidative stress and promote the production of lipid peroxides such as MDA in the gastric mucosa, resulting in oxygen free radical damage (34, 35). Conversely, our studies have demonstrated that DPHs can effectively scavenge for oxygen free radicals induced by ethanol by increasing SOD activity, and subsequently promote degradation of hydrogen peroxide, inhibit lipid peroxidation to protect the stomach from oxidation, and prevent gastric mucosal damage (36, 37).

NF-κB is a key transcription factor of inflammatory response, which mediates the entire inflammatory process. Activation of the TLR4 signaling pathway releases NF-κB from the I-κB/NF-κB complex into the cells, thus activating the expression of inflammation-related enzymes, and the production and release of downstream inflammatory cytokines, which act on the inflammatory site, in turn aggravating inflammatory response (38, 39). Therefore, NF-κB has become a crucial indicator and target for inflammatory response. Inhibiting the activation of NF-κB signaling can reduce or hinder the expansion and persistence of inflammatory response mediated by NF-κB signaling (40). In the present study, the expression of related proteins, activity of related enzymes, and the levels of inflammatory factors in gastric tissues of mice were detected. The results revealed that expression levels of TLR4, MYD88, IRAK-1, p-IκBα, and NF-κBp65 proteins in G2 gastric tissues increased considerably; an abnormal overexpression of iNOS and COX-2 was observed, and the levels of IL-1, IL-6, TNF-α, NO, and PGE2 increased significantly when compared with corresponding expression levels in G1 gastric tissues, which was consistent with the findings of previous studies (41, 42). By contrast, pre-treatment of gastric ulcers mice with DPHs decreased the expression of proteins associated with the TLR4/MYD88/NF-κB signaling pathway, decreased the activities of inflammation-related enzymes, iNOS, and COX-2, and the production and release of inflammatory factors. The results suggest that DPHs can inhibit the TLR4/MYD88/NF-κB signaling pathway, inhibit phosphorylation of the I-κB family, inhibit the release of NF-κB from the I-κB/NF-κB complex into cells, reduce the activation of related inflammatory transcription factors, and the production and release of related inflammatory factors (43-47), which, in turn, ameliorates the degree of mucosal injury and inhibits the aggravation and persistence of gastric ulcers. Specifically, the efficacy of a high dose of DPHs (G4) is similar to or superior to that of RAN.

TRPV1 also called capsaicin receptor or vanilloid receptor 1, is a non-selective cation channel located on the cell membrane and can be activated through various forms of stimulation (48, 49). A few studies have established that the expression of TRPV1 in the gastrointestinal mucosa can regulate gastric mucosal blood flow, gastric acid secretion, gastrointestinal motility, and other functions, which exert gastroprotective effects on gastric mucosal injury caused by NSAIDs and ethanol (50-52). In addition, previous studies revealed that TRPV1 was highly expressed not only in the gastric mucosa but also in submucosal blood vessels and myenteric plexus (53,54). Therefore, we believe that TRPV1 is closely associated with gastric ulcers. We evaluated the protein and mRNA expression levels of TRPV1 and related neuropeptides (CGRP and SP) to elucidate the correlation between ethanol and TRPV1 activation, and to explore whether DPHs could mediate the inhibition of TRPV1. The results revealed that ethanol stimulation increased the expression of TRPV1 and CGRP at the protein and mRNA levels in gastric tissues but decreased the expression of SP at the protein and mRNA levels, which was consistent with the findings of previous studies (53). Strikingly, pre-treatment with DPHs significantly inhibited the increase in protein and mRNA expression of TRPV1 and CGRP and promoted the protein and mRNA expressions of SP. The results suggest that DPHs are involved in the inhibition of TRPV1 response, which inhibits the excitation of neurons, thereby affecting the production and release of CGRP and SP. The events consequently terminate or reduce the occurrence and transmission of ethanol-mediated inflammatory pain (55,56), which demonstrates that blocking the activation of signaling pathways associated with TRPV1 could be one of the key mechanisms by which DPHs inhibit the persistence of gastric ulcers and enhance gastroprotective effects against ethanol-induced gastric ulcers injury.

**Table 1:** Primer sequences of related mRNA

**Primers**	**Forward/Reverse**	**Sequence**
TLR4	Forward	5'-ATGGCATGGCTTACACCACC-3'
	Reverse	5'-GAGGCCAATTTTGTCTCCACA-3'
MYD88	Forward	5'-ATCGCTGTTCTTGAACCCTCG-3'
	Reverse	5'-CTCACGGTCTAACAAGGCCAG-3'
IRAK-1	Forward	5'-ACTCCAGAGAAGTCCCAACCA-3'
	Reverse	5'-CAGGAATGCAGGGTAGCAGAG-3'
NF-κBp65	Forward	5'-TGCGATTCCGCTATAAATGCG-3'
	Reverse	5'-ACAAGTTCATGTGGATGAGGC-3'
TRPV1	Forward	5'-CCGGCTTTTTGGGAAGGGT-3'
	Reverse	5'-GAGACAGGTAGGTCCATCCAC-3'
CGRP	Forward	5'-CAGTGCCTTTGAGGTCAATCT-3'
	Reverse	5'-CCAGCAGGCGAACTTCTTCTT-3'
SP	Forward	5'-TTTCTCGTTTCCACTCAACTGTT-3'
	Reverse	5'-GTCTTCGGGCGATTCTCTGC-3'

DPHs: diphenylheptanes

DPHs: diphenylheptanes

**Table 2:** Effect of DPHs on the levels of PGE2 and NO, and the activity of COX-2 and iNOS

**Group**	**COX-2 (U/L** **)**	**PGE2 (pg/mL)**	**iNOS (U/L)**	**NO (pg/mL)**
Normal	39.55±2.66	57.91±6.61	23.04 ± 1.82	21.36 ± 2.13
RAN (100 mg/kg)	120.78±4.01 ##	267.81±15.30 ##	76.29 ± 1.96 ##	38.88 ±2.18 ##
DPHs (31.7 mg/kg)	148.58±10.38 ##	308.41±14.44 #^#^	90 ± 2.59 ##	49.04 ± 2.28 ##
DPHs (63.4 mg/kg)	123.77±7.77 ##	289.04±20.69 ^##^	76.10 ± 3.49 ##	46.96 ± 1.37 #
DPHs (126.8 mg/kg)	116.34±5 ##	283.01±9.73 ##	65.71 ± 2.01 ##	41.50 ± 2.28 ^#^
Model	263.92±18.73 ^**^	406.81±6.54 ^**^	116.93 ± 5.60 ^**^	62.19 ± 3.37 ^**^

The results showed that the mean±SEM of 6 mice in each group. ^*^P<0.05 and ^**^ *P*<0.01, compared with G1; ^#^ *P*<0.05 and ^##^ *P*<0.01, compared with G2

DPHs: diphenylheptanes; PGE2: prostaglandin E2

**Figure 7:**
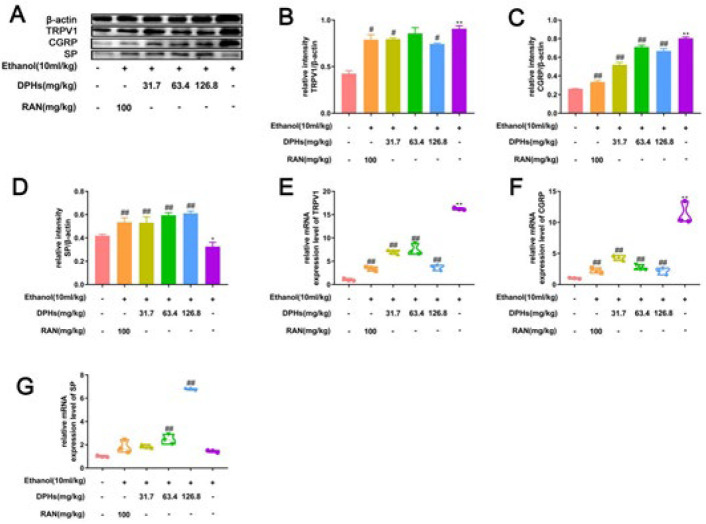
(A) Effects of DPHs on the protein expression of TRPV1, CGRP, and SP in gastric tissues. (B-D) Quantification of the relative proteins levels of TRPV1, CGRP, and SP. (E-G) Gene expression level of TRPV1, CGRP, and SP. ^**^P<0.01, compared to the G1; ^#^*P*<0.05 and^ ##^*P*<0.01, compared to the G2

TRPV1: transient receptor potential vanilloid 1; CGRP: calcitonin gene-related peptide; SP: substance P

## Conclusion

The experimental results of the present study demonstrate that DPHs exert protective effects on ethanol-induced gastric ulcers injury, and the mechanisms involved could be associated with the inhibition of the activation of TLR4/MYD88/NF-κB and TRPV1 signaling pathways, inhibition of the release of downstream inflammatory factors, reduction of oxidative stress, and enhancement of gastrointestinal motility. The results provide a theoretical basis for the anti-gastric ulcers effect of *A. officinarum* and demonstrate the gastroprotective effects of DPHs extracted from *A. officinarum* rhizomes. Therefore, DPHs from *A. officinarum* rhizomes can be used to manufacture pharmaceutical supplements, which present great potential in the treatment of various aspects of health status and resistance to diseases caused by external stress, especially, in the prevention and improvement of ethanol-induced gastric ulcers. 
